# Purpuric lesions and atrophic scars in a neonate

**DOI:** 10.1016/j.jdcr.2021.01.006

**Published:** 2021-01-15

**Authors:** Lynette Wei-Yi Wee, Woei-Kang Liew, Mark Jean-Aan Koh

**Affiliations:** a Dermatology Service, KK Women's & Children's Hospital, Singapore, Singapore; b Rheumatology and Immunology Service, Department of Paediatric Subspecialties, KK Women's & Children's Hospital, Singapore, Singapore

**Keywords:** atrophic scars, auto-antibodies, neonatal lupus, EM, erythema multiforme, JXG, juvenile xanthogranulomas, LCH, langerhans cell histiocytosis, NLE, neonatal lupus erythematosus

A 61-day-old healthy boy, born at full term to non-consanguineous healthy parents, presented to the pediatric dermatology clinic for a 4-week history of generalized, discrete, asymptomatic erythematous papules over his scalp, face, trunk, limbs, palms, and soles (Fig 1, *A*), which had evolved into annular scaly plaques over his face a week before (Fig 1, *B*). He had a good birthweight and an unremarkable antenatal history, including negative maternal syphilis and hepatitis B antibody tests. He also had three hyperpigmented atrophic scars over the midline of his back that were present from birth (Fig 2). A punch biopsy was performed on a thigh lesion (Fig 3).

**Fig 1:**
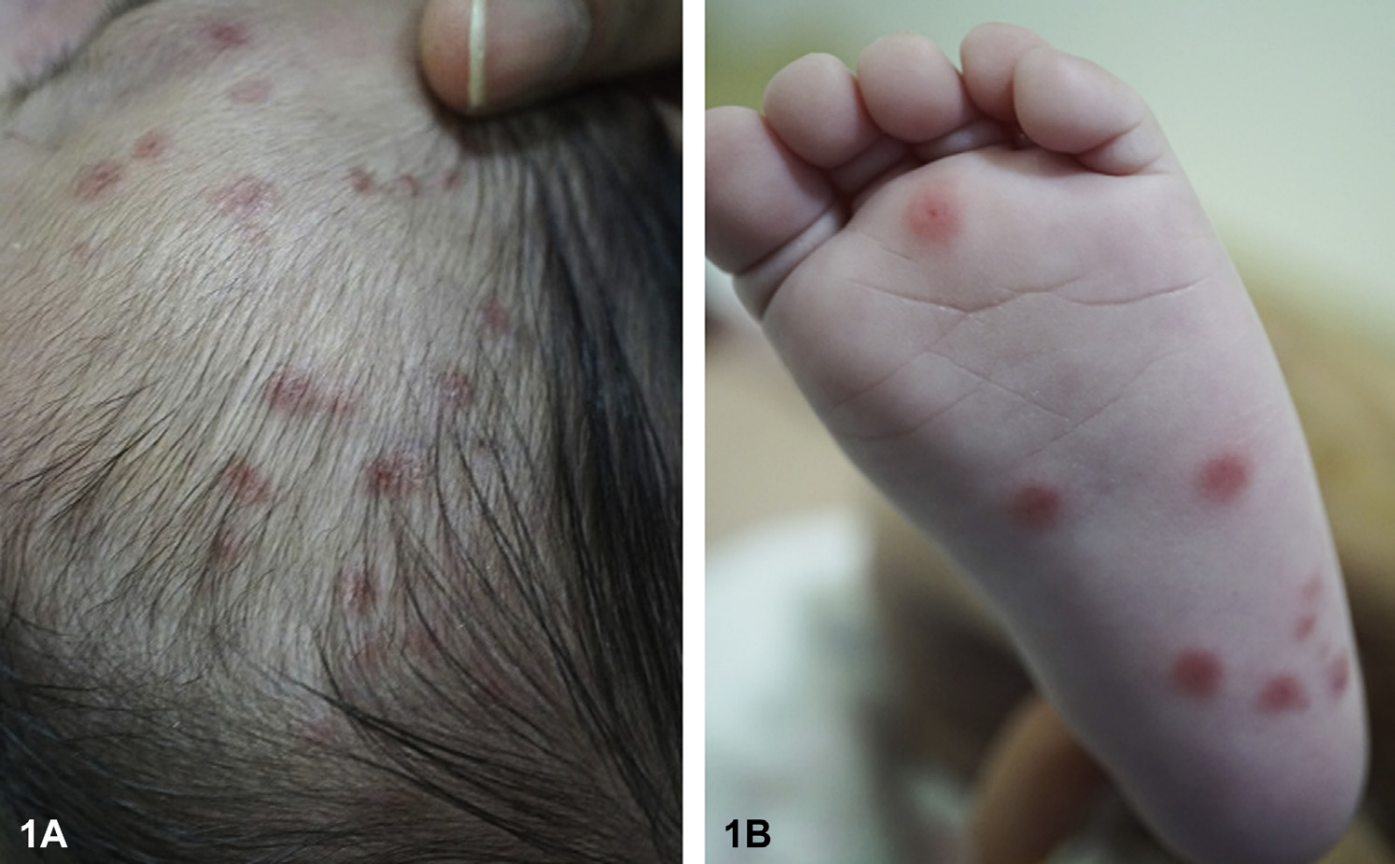

**Fig 2:**
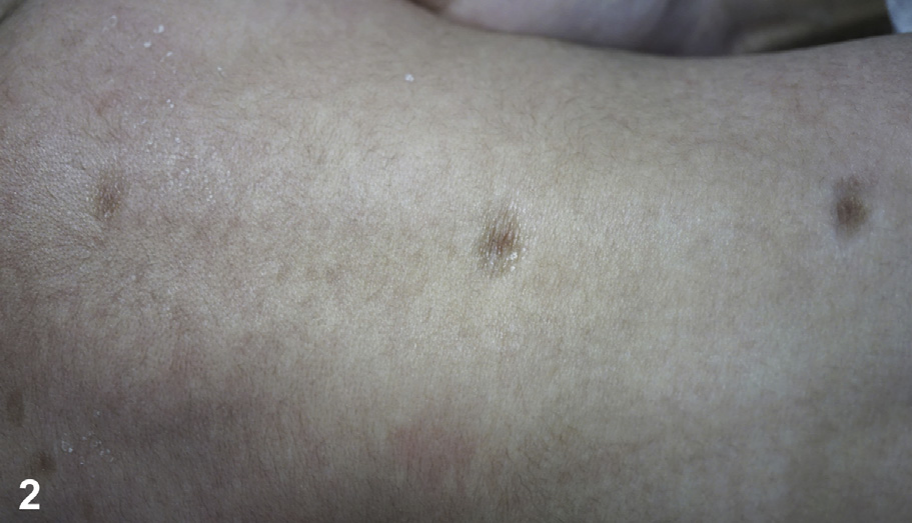

**Fig 3:**
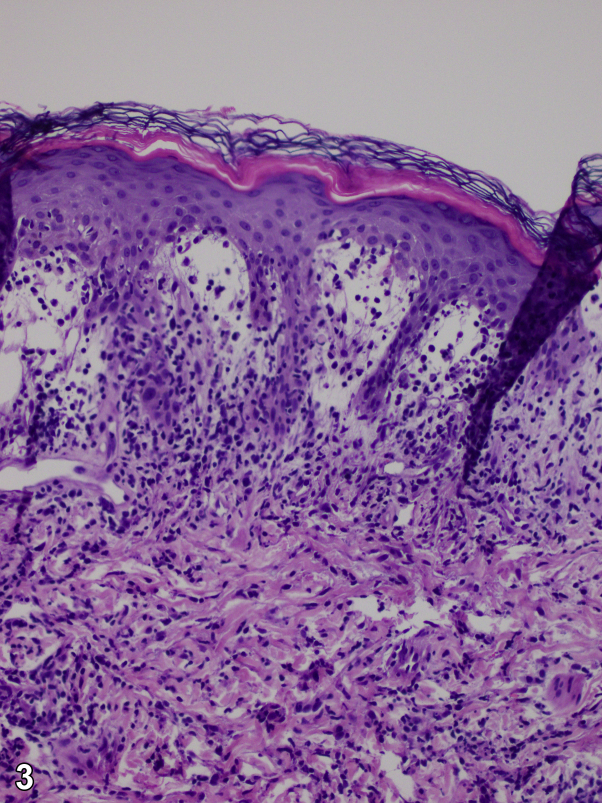

**Question 1: Which of the following histologic findings do you see in this patient's biopsy in** Fig 3**?**

A. The dermis showed aggregates of large mononuclear histiocytes with reniform, irregular, cleaved nuclei and abundant eosinophilic cytoplasm with surrounding eosinophils diffusely scattered
B. Psoriasiform hyperplasia with superficial neutrophils with numerous plasma cells and endothelial swelling
C. Dense, foamy lymphohistiocytic proliferation of the dermis with Touton giant cells
D. Vacuolar interface dermatitis showing a lymphocytic infiltrate along the dermoepidermal junction associated with hydropic changes and dyskeratosis of basal keratinocytes
E. Basal vacuolar alteration at the dermal-epidermal junction, with superficial and deep perivascular and periadnexal lymphohistiocytic infiltrates

**Answers:**

A. The dermis showed aggregates of large mononuclear histiocytes with reniform, irregular, cleaved nuclei and abundant eosinophilic cytoplasm with surrounding eosinophils diffusely scattered–Incorrect.
B. Psoriasiform hyperplasia with superficial neutrophils with numerous plasma cells and endothelial swelling–Incorrect.
C. Dense, foamy lymphohistiocytic proliferation of the dermis with Touton giant cells–Incorrect.
D. Vacuolar interface dermatitis showing a lymphocytic infiltrate along the dermoepidermal junction associated with hydropic changes and dyskeratosis of basal keratinocytes–Incorrect.
E. Basal vacuolar alteration at the dermal-epidermal junction, with superficial and deep perivascular and periadnexal lymphohistiocytic infiltrates–Correct.

**Question 2: What is the most likely diagnosis?**

A. Congenital syphilis
B. Multiple juvenile xanthogranulomas (JXG)
C. Neonatal lupus erythematosus (NLE)
D. Langerhans cell histiocytosis (LCH)
E. Erythema multiforme (EM)

**Answers:**

A. Congenital syphilis–Incorrect. Congenital syphilis, caused by the spirochete, treponema pallidum, is acquired by the fetus *in utero*. Symptoms of early congenital syphilis include fever, rashes, and low birth weight. Rashes in early congenital syphilis are classically vesiculobullous or maculopapular, occurring on the palms and soles, and may be associated with desquamation. Diagnosis is confirmed by positive treponemal and non-treponemal serology in the mother or baby. Our patient's mother had negative syphilis serology during pregnancy, and the child did not show other signs of congenital syphilis. Typical histological features include psoriasiform hyperplasia with superficial neutrophils with numerous plasma cells and endothelial swelling.^1^
B. Multiple JXG–Incorrect. JXG is the most common form of cutaneous non-Langerhans cell histiocytosis. It is a benign condition typically occurring during infancy or early childhood and characterized clinically by small, yellowish-brown papules. The morphology of this patient's rash is not typical of JXG, although atypical variants of JXG could still be considered, underlying the importance of dermatopathological correlation. Early JXG lesions exhibit histological features of a dense lymphohistiocytic proliferation in the dermis with multiple foamy histiocytes and Touton giant cells, while in late JXG lesions, histological features include short fascicles of fibrohistiocytic cells and fibrosis.^2^
C. NLE–Correct. NLE is an uncommon condition caused by the transplacental transfer of maternal autoantibodies, most commonly anti-Ro (anti-SSA) and anti-La (anti-SSB) antibodies. The incidence is unknown as many cases go undetected. Skin manifestations are common and usually presents after a few weeks of life with the classical scaly, annular rash in a photo-sensitive distribution. Sun or ultraviolet light exposure is known to precipitate the lesions of NLE. The most common sites of involvement are the scalp, face, arms, and legs, with truncal and groin areas less often involved. Congenital lesions are uncommon and are present in about 15% of cases, with atrophic lesions being rarely reported. Basal vacuolar alteration at the dermal-epidermal junction, with superficial and deep perivascular and periadnexal lymphohistiocytic infiltrates are the typical histological features. NLE is an uncommon condition caused by the transplacental transfer of maternal autoantibodies, most commonly anti-Ro (anti-SSA) and anti-La (anti-SSB) antibodies. Diagnosis is confirmed with positive autoantibodies. In this case, baby and mother's anti-Ro (SSA) antibodies returned positive at 5.6 and 6.1 units respectively (normal <1.0 unit). Antinuclear antibody tests were also positive at titers of 1:160 in the baby and 1:640 in the mother. The child's complete blood count and electrocardiogram were also normal. He was diagnosed with NLE. NLE most commonly presents after a few weeks of life with the classical annular rash in photodistributed sites. Atrophic scarring is an uncommon presentation of NLE, and is rarely seen except in congenital lesions. While most mothers are asymptomatic, they should be monitored for Sjögren syndrome or systemic lupus erythematosus, which may develop subsequently.^3^^,^^4^
D. LCH–Incorrect. LCH is an inflammatory myeloid neoplasia associated with mutations of several genes in the mitogen-activated protein kinase pathway. Skin involvement is most commonly characterized by an extensive seborrheic dermatitis-like rash on the scalp, mimicking persistent cradle cap; an erythematous papular rash similar to *Candida* diaper rash; or deep ulcerative lesions in the groin or axilla, and purplish-brown lesions. The relatively acute presentation of this patient makes LCH less likely, although atypical variants of LCH could still be considered, underlying the importance of dermatopathological correlation. The typical histological finding in LCH includes dermal aggregates of large mononuclear histiocytes with reniform, irregular, cleaved nuclei and abundant eosinophilic cytoplasm with surrounding eosinophils diffusely scattered.^5^
E. EM–Incorrect. EM is extremely rare in the infantile period. The morphology of the subsequent annular rash in our case is suggestive of EM, but the history of initial papules is atypical. Additionally, compared with NLE, EM does not usually occur more prominently over photodistributed areas. Suggested etiologies include cow's milk protein or drug allergy, hepatitis B vaccination, and *Candida* infection. Vacuolar interface dermatitis, showing a lymphocytic infiltrate along the dermoepidermal junction associated with hydropic changes and dyskeratosis of basal keratinocytes, is the typical histological feature.

**Question 3: What further evaluation is recommended?**

A. Abdominal ultrasound
B. Skeletal survey
C. Detailed ophthalmological assessment
D. Full blood count and 12-lead electrocardiogram
E. Magnetic resonance imaging of the brain

**Answers:**

A. Abdominal ultrasound–Incorrect. Although hepatic involvement has been reported in NLE, it is not the most common complication. The elevations in liver enzymes, if present, are usually transient and generally resolve within the first months of post-natal life without sequelae. An abdominal ultrasound will not be useful in the evaluation of liver involvement.
B. Skeletal survey–Incorrect. Chondrodysplasia punctata is a rare condition characterized by radiographic evidence stippling of the epiphyses and/or the spine and has been reported in NLE, although it is not specific to NLE and can also occur with other disorders, such as genetic disorders, fetal viral exposure, and the use maternal medications including dilantin and warfarin. It generally resolves without treatment during childhood within the first year and, unless clinically indicated, is not the most urgent item to screen for in a case of suspected NLE.
C. Detailed ophthalmological assessment–Incorrect. Eye complications have not been reported in NLE.
D. Full blood count and 12-lead electrocardiogram–Correct. Hematological complications of NLE have been well reported. Infants may have thrombocytopenia or neutropenia. Although transient, severe thrombocytopenia can result in bleeding. More rarely, a hemolytic anemia or even a pancytopenia or aplastic anemia can occur. Although thrombocytopenia and hepatitis are usually transient, congenital heart block is permanent and requires treatment with pacemakers.
E. Magnetic resonance imaging of the brain–Incorrect. Hydrocephalus secondary to NLE have been uncommonly reported, typically occurring between 12 and 24 months. In the absence of neurological signs and symptoms or increasing occipital-frontal-circumference or bulging fontanelles, magnetic resonance imaging of the brain, which is usually performed under moderate sedation or general anesthesia in young infants is not the most recommended evaluation.

## Conflicts of interest

None disclosed.
